# Factors Associated with Hospital Geriatric Service Availability Beyond Urban-Rural Differences

**DOI:** 10.1093/geroni/igaf122.3339

**Published:** 2025-12-31

**Authors:** Teagan Maguire, Alice Yan, Jie Chen

**Affiliations:** University of Maryland College Park, College Park, Maryland, United States; University of Maryland College Park, College Park, Maryland, United States; University of Maryland College Park, College Park, Maryland, United States

## Abstract

The study examined variations in the provision of geriatric care among hospitals based on their urban and rural locations. This cross-sectional study utilized data from the 2023 American Hospital Association Annual Survey and the Area Health Resources File. Descriptive statistics and logistic regression models were employed to compare hospital and geographic variations among hospitals with or without geriatric services. Geriatric services were defined as offering one or more of the following: adult day care, Alzheimer’s diagnostic-assessment services, comprehensive geriatric assessment, emergency response systems, geriatric acute care units, and/or geriatric clinics. The study included 2,991 hospitals, categorized as metropolitan (61.82%), micropolitan (16.85%), and rural (21.33%). Descriptive analyses indicate that hospitals in rural areas provide fewer geriatric services. After controlling for hospital size, ownership type, teaching affiliation, and community demographics, hospitals in metropolitan areas were significantly less likely to provide hospital-based geriatric services (OR = 0.68, 95% CI: 0.51–0.91) or offer geriatric psychiatry services (OR = 0.60, 95% CI: 0.39–0.94), compared to rural hospitals. Hospitals with larger bed sizes, teaching hospital status, and locations in areas with more skilled nursing facilities were more likely to provide geriatric services. However, hospitals serving higher proportions of minority populations were significantly less likely to offer geriatric services. A similar pattern was observed among hospitals providing Alzheimer’s services and geriatric psychiatric care. Results indicate that differences in geriatric service provision between urban and rural hospitals are largely explained by hospital characteristics, local resources, and population demographics rather than location alone.

